# Screening for marginal food security in young children in primary care

**DOI:** 10.1186/s12887-021-02674-4

**Published:** 2021-04-23

**Authors:** Imaan Bayoumi, Catherine S. Birken, Kimberly M. Nurse, Patricia C. Parkin, Jonathon L. Maguire, Colin Macarthur, Janis A. Randall Simpson, Cornelia M. Borkhoff

**Affiliations:** 1 Department of Family Medicine, Queen’s University, 220 Bagot St., P.O. Bag 8888, Kingston, ON K7L5E9 Canada; 2 Centre for Studies in Primary Care, Queen’s University, Kingston, ON Canada; 3 Division of Paediatric Medicine and the Paediatric Outcomes Research Team (PORT), Hospital for Sick Children, Toronto, ON Canada; 4 Institute of Health Policy, Management and Evaluation, University of Toronto, Toronto, ON Canada; 5 Sick Kids Research Institute, Toronto, ON Canada; 6 Department of Pediatrics, Faculty of Medicine, University of Toronto, Toronto, ON Canada; 7 Li Ka Shing Knowledge Institute, St. Michael’s Hospital, Toronto, ON Canada; 8 Department of Paediatrics, St. Michael’s Hospital, Toronto, ON Canada; 9 Department of Nutritional Sciences, Faculty of Medicine, University of Toronto, Toronto, ON Canada; 10 Department of Family Relations and Applied Nutrition, University of Guelph, Guelph, ON Canada

## Abstract

**Background:**

Household food insecurity (FI), even at marginal levels, is associated with poor child health outcomes. The Nutrition Screening Tool for Every Preschooler (NutriSTEP®) is a valid and reliable 17-item parent-completed measure of nutrition risk and includes a single item addressing FI which may be a useful child-specific screening tool. We evaluated the diagnostic test properties of the single NutriSTEP® FI question using the 2-item Hunger Vital Sign™ as the criterion measure in a primary care population of healthy children ages 18 months to 5 years.

**Results:**

The sample included 1174 families, 53 (4.5%) of which were marginally food secure. An affirmative response to the single NutriSTEP® question “I have difficulty buying food I want to feed my child because food is expensive” had a sensitivity of 85% and specificity of 91% and demonstrated good construct validity when compared with the Hunger Vital Sign™.

**Conclusion:**

The single NutriSTEP® question may be an effective screening tool in clinical practice to identify marginal food security in families with young children and to link families with community-based services or financial assistance programs including tax benefits.

**Trial registration:**

*TARGet Kids!* practice-based research network (Registered June 5, 2013 at www.clinicaltrials.gov; NCT01869530); www.targetkids.ca

## Introduction

Household food insecurity (FI) — a household’s experience of inadequate or insecure access to sufficient, safe and nutritious food because of income or finances — is a major public health problem [1, 2]. Household food insecurity is associated with poor child health outcomes, even at marginal levels [3–5]. Previous research also shows that families with marginal food security are more like food-insecure households than food-secure households [6].

Families affirming one or both of the first 2 items of the 18-item U.S. Household Food Security Screening Module (HFSSM), are considered marginally food secure [6, 7]. The first item measures uncertainty about having enough food and the second item measures uncertainty about exhausting their food supply. Developed in the U.S., these items comprise the 2-item Hunger Vital Sign™ (HVS) [8], now advocated for use as a screening tool for marginal food security in clinical practice and embedded into the electronic medical record system along with clinical and billing codes in some areas. However, the 2 items on the HVS™ are from the adult module in the 18-item HFSSM, which may not apply to children [9]. Furthermore, a 1-item child-specific screen for use in paediatric primary care practice may have greater utility in identifying marginal food security in families with young children.

The Nutrition Screening Tool for Every Preschooler (NutriSTEP®) is a valid and reliable 17-item parent-completed questionnaire developed in Canadian children addressing multiple domains of nutrition risk [10, 11]. A single item addressing FI may be a useful child-specific screening tool. The 17-item NutriSTEP® has an area under the curve of 84.6% compared with a dietitian-completed assessment [10, 11]. However, the accuracy of the FI question is unknown. We aimed to examine the diagnostic test properties of the single NutriSTEP® FI question.

## Methods

This cross-sectional study enrolled healthy children 18 months to 5 years of age during scheduled health supervision visits at primary care practices in Toronto, Canada participating in a research network called TARGet Kids! (www.targetkids.ca). TARGet Kids! is an ongoing, open, longitudinal cohort enrolling healthy children from birth to age 5 years. The profile of this cohort has been previously described [12]. Study participants were recruited by trained research personnel embedded in participating practices. Informed consent was obtained from parents of participants, who completed standardized questionnaires including the FI screens. For the purpose of this study, children were included if they had complete data on both the HVS™ and NutriSTEP® questionnaires. All methods were performed in accordance with the relevant guidelines and regulations and approved by Research Ethics Boards at the Hospital for Sick Children and St. Michael’s Hospital, Toronto.

Parents completed the HVS™, the first 2 items of the 18-item HFSSM; this brief 2-item measure has 97% sensitivity and 83% specificity for identifying marginal food security, using the HFSSM as the criterion measure [8]. The HVS™ questions are: “Within the past 12 months, we worried whether our food would run out before we got money to buy more” and “Within the past 12 months, the food we bought just didn’t last and we didn’t have money to buy more” with response options “Was that often true, sometimes true or never true for your household in the past twelve months?” According to convention, both items were coded as an affirmative response if either “often true” or “sometimes true” was selected [7, 8]. By definition, families affirming one or both items were classified as having marginal food security.

Parents also completed the 17-item NutriSTEP® which included the FI question: “I have difficulty buying food I want to feed my child because food is expensive” with response options: “most of the time”; “sometimes”; “rarely”; “never”. Using the same convention as for the HVS™ questions, this single item was coded as an affirmative response if “most of the time”, “sometimes”, or “rarely” was selected. We included “rarely” as an affirmative response as we reasoned that families struggling to meet their needs, may either choose not to disclose or select “rarely” due to stigma or shame [13]. Families affirming this single item were classified as having marginal food security.

We examined the diagnostic test properties (including sensitivity and specificity) of the 1-item NutriSTEP® FI question using the 2-item HVS™ as the criterion measure. We then examined convergent construct validity (the correspondence between the 1-item NutriSTEP® screen and theoretically related variables, e.g. self-report family income) using multiple logistic regression. Convergent construct validity was tested by comparing the multiple logistic regression models for the 1-item NutriSTEP® with those of the 2-item HVS™ evaluating the associations of these FI measures with variables considered predictors of marginal food security (family income, maternal education and parent employment status) adjusting for covariates. Missing covariate data (< 15% missing) were handled using multiple imputation. Statistical significance was defined as *p* < 0.05, and all statistical tests were 2-sided. Statistical analysis was conducted using SAS version 9.4 (SAS Institute).

## Results

### Participants

Of 1753 children recruited to the TARGet Kids! cohort, 1174 children had complete data on the HVS™ and NutriSTEP® questionnaires. Of these, 53 (4.5%) children were living in families with marginal food security as determined by the 2-item HVS™. Compared with families not at risk for food insecurity, a higher proportion of children in families at risk for food insecurity had a lower family income, a mother with high school education or less, 1 or both parents were unemployed, and were from a single-parent family (Table 1).

**Table 1:** Characteristics of 1174 study participants by marginal food security status (as determined by the 2-item Hunger Vital Sign™)

Characteristic	All Participants		Marginal food security	
			Yes	No
N	n^b^	1174	53 (4.5)	1121 (95.5)
Age, months	1174	37 (24–50)	35 (18–65)	37 (18–65)
Female sex	1174	552 (47.1)	27 (50.9)	525 (46.8)
Maternal ethnicity^a^	991			
African and Caribbean		32 (3.2)	6 (13.0)	26 (2.8)
Asian		171 (17.3)	11 (23.9)	160 (16.9)
European		677 (68.3)	20 (43.5)	657 (69.5)
Indigenous		5 (0.5)	0 (0.0)	5 (0.5)
Latin American		35 (3.5)	3 (6.5)	32 (3.4)
Mixed		71 (7.2)	6 (13.0)	65 (6.9)
Maternal education	1148			
High School or less		75 (6.5)	22 (42.3)	53 (4.8)
College/Trades Diploma		151 (13.2)	12 (23.1)	139 (12.7)
University Degree		922 (80.3)	18 (34.6)	904 (82.5)
Self-report family income (CAN $)	1123			
Less than $40,000		97 (8.6)	25 (50.0)	72 (6.7)
$40,000 - $79,999		149 (13.3)	14 (28.0)	135 (12.6)
$80,000 - $149,999		352 (31.3)	10 (20.0)	342 (31.9)
$150,000 +		525 (46.8)	1 (2.0)	524 (48.8)
Parent employment status	1099			
Both parents unemployed		20 (1.8)	9 (17.7)	11 (1.1)
1 parent employed		245 (22.3)	17 (33.3)	228 (21.8)
2 parents employed		834 (75.9)	25 (49.0)	809 (77.2)
Family living arrangements	1168			
Single parent or other household		63 (5.4)	14 (26.4)	49 (4.4)
2 parents in the same household		1105 (94.6)	39 (73.6)	1066 (95.6)
Family immigration status	1008			
Non-Immigrant (CDN born)		573 (56.9)	19 (45.2)	554 (57.4)
Immigrant, industrialized		166 (16.5)	6 (14.3)	160 (16.6)
Immigrant, non-industrialized		269 (26.7)	17 (40.5)	252 (26.1)

Data are presented as n (%) or median (IQR)

^a^Maternal ethnicity: European includes Western European, Eastern European, Australian or New Zealander; Asian includes East Asian, Southeast Asian, South Asian, West Asian/North African; African and Caribbean, Latin American, Indigenous, Mixed = 2 or more ethnic groups

^b^This column shows the distribution of participant characteristics for the response sample

### Diagnostic test properties of the 1-item NutriSTEP®

The diagnostic test properties of the single FI NutriSTEP® question, compared with the 2-item HVS™ as the criterion measure were: sensitivity 84.9% (95% CI: 72.4, 93.3), specificity 91.2% (95% CI: 89.4, 92.8), false positive rate 8.8% (95% CI: 7.2, 10.8), positive predictive value 31.3% (95% CI: 26.7, 36.2), negative predictive value 99.2% (95% CI: 98.5, 99.6). An affirmative response indicating marginal food security on the 1-item screen was 9.6 times more likely (LR+ = 9.6; 95% CI: 7.7, 12.0) for those with marginal food security as determined by the 2-item HVS™ (Table 2).

**Table 2:** Diagnostic test properties of marginal food security status based on the 1-item NutriSTEP® question compared with the 2-item Hunger Vital Sign™

1-item NutriSTEP®	2-item hunger vital sign™		
	Yes	No	Total
**Yes**	45	99	144
**No**	8	1022	1030
**Total**	53	1121	1174
**Sensitivity:** 45 / (45 + 8) * 100 **= 84.9%** (95% CI: 72.4, 93.3)			
**Specificity:** 1022 / (1022 + 99) * 100 **= 91.2%** (95% CI: 89.4, 92.8)			
**PPV:** 45 / (45 + 99) * 100 = **31.3%** (95% CI: 26.7, 36.2)			
**NPV:** 1022 / (1022 + 8) * 100 = **99.2%** (95% CI: 98.5, 99.6)			
**Likelihood Ratio (for a positive test):** sensitivity / (1-specificity) = **9.6** (95% CI: 7.7, 12.0)			
**Likelihood Ratio (for a negative test):** (1 – sensitivity / specificity) = **0.2** (95% CI: 0.1, 0.3)			
**Accuracy:** sensitivity x prevalence + specificity x (1 – prevalence) = **90.9%** (95% CI: 89.1, 92.5)			

### Construct validity

An affirmative response to the single NutriSTEP® FI question was associated with higher odds of family income less than CAD $40,000 (adjusted odds ratio [aOR]: 7.4; 95% CI: 3.4, 16.1; *p* < 0.001), lower maternal education (high school or less, aOR: 1.8; 95% CI: 0.9, 3.7; *p* = 0.09; college/trades certificate, aOR: 1.7; 95% CI: 1.0, 2.8; *p* = 0.049), and both parents being unemployed (aOR: 3.3; 95% CI: 1.1, 10.4; p = 0.04). These associations were similar to (perhaps somewhat weaker than) the corresponding associations for the HVS™ (Table 3).

**Table 3:** Relation between marginal food security based on the 2-item Hunger Vital Sign™ and on the 1-item NutriSTEP® question with family income, maternal education, and parent employment status (*N* = 1174)

Outcome	2-item Hunger Vital Sign™		1-item NutriSTEP®	
	Marginal food security = ***yes***		Marginal food security = ***yes***	
	aOR (95% CI)	*p*	aOR (95% CI)	*p*
**Self-report family income**^a^				
Less than $40,000	21.77 (3.92, 121.01)	< 0.001	7.43 (3.44, 16.05)	< 0.001
$40,000 - $79,999	15.77 (2.94, 84.67)	0.001	6.34 (3.40, 11.83)	< 0.001
$80,000 - $149,999	6.23 (1.17, 33.26)	0.03	2.66 (1.53, 4.63)	< 0.001
$150,000 +	1.00		1.00	
**Maternal education**^b^				
High School or less	4.75 (1.95, 11.58)	< 0.001	1.82 (0.90, 3.68)	0.09
College/Trades Diploma	1.75 (0.76, 4.04)	0.19	1.68 (1.00, 2.82)	0.049
University Degree	1.00		1.00	
**Parent employment status**^c^				
Both parents unemployed	6.26 (1.72, 22.81)	0.006	3.31 (1.06, 10.35)	0.04
1 parent employed	0.83 (0.39, 1.79)	0.64	0.95 (0.59, 1.53)	0.83
2 parents employed	1.00		1.00	

^a^ Model adjusted for age, sex, maternal ethnicity, maternal education, parent employment status

^b^ Model adjusted for age, sex, maternal ethnicity, family income, parent employment status,

^c^ Model adjusted for age, sex, maternal ethnicity, maternal education, family income,

## Discussion

In this large healthy child cohort recruited in urban Canadian primary care practice, the 1-item NutriSTEP® FI question demonstrated strong diagnostic test properties and good construct validity. This single question may be an effective screening tool for identifying young children living in families with marginal food security in primary care settings. Furthermore, increasingly recognized is the importance of nutrition security, defined as ‘having consistent access, availability, and affordability of foods and beverages that promote well-being and prevent disease’ [14]. When using the 17-item NutriSTEP® in healthy toddlers and preschoolers in community settings, this single question provides clinicians with a valid measure of marginal food security, as well as nutrition risk.

Marginal household food security is associated with poor educational outcomes and emotional and behavioural problems in children, as well as maternal major depression and anxiety [3–5]. Sociodemographic characteristics of households reporting marginal food security (affirming 1 or 2 items) are more similar to those experiencing more severe food insecurity (affirming > 2 items) than food secure households (affirming 0 items) [3–5]. Such evidence of associations of marginal food security and both immediate and long-term adverse health outcomes in young children highlights the importance of screening for social needs in paediatric primary care practice in an empathic and efficient manner.

There are study limitations. First, we did not use the 18-item HFSSM as our criterion measure, so we could not examine associations between the single question and more severe household FI. However, because we were evaluating a brief measure of marginal food security suitable for screening in healthcare settings, the HVS™ is considered the gold standard and therefore is the more appropriate criterion measure. In addition, detection of marginal food security is an appropriate target for a clinical screening tool. Second, our sample had a relatively high family income, which may not be representative of other families. However, because FI screening tools are likely to perform better in low income populations, it is important to evaluate them in an economically diverse population [9].

The 1-item NutriSTEP® FI question is an alternative brief measure of marginal food security and one that is child-specific, which is suitable for screening for marginal food security in families with children in clinical settings. While no previous study has examined this single item on the NutriSTEP® as a food security screen, others have examined the validity of a single item measure of food security. Nolan et al. [15] validated the single question “In the past 12 months, were there any times that you ran out of food and couldn’t afford to buy more?” using the HFSSM as the criterion measure in a random sample of households in three low income regions in Australia, including 56% with children under age 18 years. The question had high specificity (96%) but low sensitivity (56.9%). Urke et al. examined each question in the 18-item HFSSM with the purpose of developing a rapid assessment of food security among Inuit adults and children. They identified one child item (“In the last 12 months, were there times when it was not possible to feed the children a healthy meal because there was not enough money?”) with strong diagnostic test properties using an affirmative response to any 2 HFSSM questions as their criterion measure [9]. Our study differs from Urke et al. in several ways. Our study was conducted in an urban primary care setting, with an anticipated lower prevalence of FI than seen in the remote Arctic setting. In addition, we used the HVS™ as the criterion measure, a measure of marginal food security, rather than the more severe problem of food insecurity. It is possible that the 1-item NutriSTEP® FI question (“I have difficulty buying food I want to feed my child because food is expensive”) may more effectively target marginal food security, which is a more appropriate target for primary care screening efforts. Future research should empirically examine this hypothesis.

While healthcare providers recognize the importance of identifying poverty in clinical settings, they identify time constraints and multiple competing demands as barriers to integrating social needs screening into healthcare [16]. Using a single question to measure marginal food security may be more feasible than using a 2-item tool in a busy practice and would allow clinicians to intervene on unmet social needs by linking families to community-based services or financial assistance programs including tax benefits to which they may be entitled. Despite the clear importance of social determinants to child health, limited research has addressed clinical implementation of social needs screening. However, the importance of identification of caregiver needs and priorities, and referral to appropriate community supports have been highlighted [17, 18]. Furthermore, caregivers experiencing food insecurity report feeling ashamed or embarrassed in reporting FI and that health care provider empathy, concern and empowerment can mitigate these challenges [13]. This single question may be useful for opening a dialogue in the context of a trusting relationship with a health care provider, thus facilitating linkage of families with needed resources.

## Conclusion

The single NutriSTEP® question may be an effective screening tool in clinical practice to identify marginal food security in families with young children and to link families with community-based services or financial assistance programs including tax benefits.

## Data Availability

Data are available upon request by contacting www.targetkids.ca/contact-us/. The full data are not freely available to respect the confidentiality of our participants, ensure data integrity, and avoid scientific overlap between projects. Once initial contact has been made, we request a short research proposal which will be subject to review by the TARGet Kids! Scientific Committee and approval by institutional IRBs.

## Abbreviations

CI: Confidence Interval
NHQ: Nutrition and Health Questionnaire
OR: Odds Ratio
TARGet Kids!: The Applied Research Group for Kids
